# Phytobiotics to improve health and production of broiler chickens: functions beyond the antioxidant activity

**DOI:** 10.5713/ab.20.0842

**Published:** 2021-02-14

**Authors:** Motoi Kikusato

**Affiliations:** Animal Nutrition, Life Sciences, Graduate School of Agricultural Science, Tohoku University, Sendai 980-8572, Japan

**Keywords:** Herbs, Polyphenols, Growth Promotion, Molecular and Cellular Mechanism, Inflammation, Microbiota

## Abstract

Phytobiotics, also known as phytochemicals or phytogenics, have a wide variety of biological activities and have recently emerged as alternatives to synthetic antibiotic growth promoters. Numerous studies have reported the growth-promoting effects of phytobiotics in chickens, but their precise mechanism of action is yet to be elucidated. Phytobiotics are traditionally known for their antioxidant activity. However, extensive investigations have shown that these compounds also have anti-inflammatory, antimicrobial, and transcription-modulating effects. Phytobiotics are non-nutritive constituents, and their bioavailability is low. Nonetheless, their beneficial effects have been observed in several tissues or organs. The health benefits of the ingestion of phytobiotics are attributed to their antioxidant activity. However, several studies have revealed that not all these benefits could be explained by the antioxidant effects alone. In this review, I focused on the bioavailability of phytobiotics and the possible mechanisms underlying their overall effects on intestinal barrier functions, inflammatory status, gut microbiota, systemic inflammation, and metabolism, rather than the specific effects of each compound. I also discuss the possible mechanisms by which phytobiotics contribute to growth promotion in chickens.

## INTRODUCTION

### What are phytobiotics?

Phytobiotics, also referred to as phytochemicals or phytogenics, are a broad subset of plant-derived bioactive compounds. Thus far, more than 5,000 individual dietary phytobiotics have been identified in fruits, vegetables, whole grains, legumes, nuts, herbs, and essential oils [1]. Phytobiotics have also been defined as non-nutritive compounds and are therefore distinguished from the nutrients found in plants, such as vitamins and minerals. Phytobiotics can be divided into the following six categories: phenolic compounds, alkaloids, nitrogen-containing compounds, organosulfur compounds, phytosterols, and carotenoids, and they are further divided into several subcategories [1]. There have been several investigations on phenolic compounds and carotenoids to determine their biological effects and characteristics [2–5].

Phytobiotics are synthesized by plants to offer protection against invasive pathogens such as bacteria, viruses, and fungi. They also protect DNA and photosynthetic apparatus from oxidative damage in plants caused by ultraviolet radiation. Oxidative stress occurs when the formation of reactive oxygen species (ROS) exceeds the cellular antioxidant capacity, which is regulated by antioxidant enzymes, such as superoxide dismutase (SOD), catalase (CAT), and glutathione peroxidase; low-molecular constituents such as tocopherols, ascorbic acid, uric acid, imidazole dipeptides, and bilirubin; proteins that chelate free transition metals; ovotransferrin; and ceruloplasmin. The antioxidant activity of phytobiotics, especially phenolic acids and flavonoids, is predominantly determined by the structure and electron delocalization over an aromatic nucleus [6]. It has also been proposed that polyphenolic compounds exhibit antioxidant effects through a variety of mechanisms, rather than the single mode of action of typical synthetic antioxidants [7,8]. The beneficial effects of phytobiotics are considered to be due to their antioxidant properties. However, several studies have demonstrated that the antioxidant properties alone cannot explain these benefits [9,10]. In this regard, the concentration of phytobiotics and their metabolites detected in the plasma and tissues after gavage has been found to be more than 50-fold lower than that of endogenous antioxidants, such as uric acid and bilirubin [11].

In the last two decades, phytobiotics have been shown to exert multiple effects, including anti-inflammatory, antimicrobial, anti-oxidative, and metabolic-modulating effects [10,12,13]. Phytobiotics are used for promoting growth and improving meat and egg quality in poultry production [14–17]. Moreover, as antimicrobial/antibiotic growth promoters (AGPs) have been gradually eliminated from animal production owing to the increased risk of occurrence of antimicrobial resistance, the use of phytobiotics as an alternative to AGPs has been extended to farm animals for improving their intestinal status and subsequently promoting growth [18,19]. A few reviews have suggested the possible mechanisms by which phytobiotics lead to health benefits and growth promotion [20,21]. In this review, I focused on the bioavailability of phytobiotics and the possible mechanisms underlying their overall effects on intestinal barrier functions, inflammatory status, gut microbiota, systemic inflammation, and metabolism, rather than the specific effects of each compound. I also discuss the possible mechanisms by which phytobiotics contribute to growth promotion in chickens. We entirely used data from studies using laboratory mammals or cell culture, as there are a limited number of studies on the precise mechanism of phytobiotics in chickens. Nevertheless, I believe that this paper will contribute to further our understanding of the precise mechanism of the effects of phytobiotics.

## BIOAVAILABILITY

Bioavailability is the fraction (%) of an administered compound/drug that reaches the systemic circulation following ingestion into the gastrointestinal tract. The concentration of phytobiotics and their metabolites in the blood and tissues is very low; only 2% to 15% of phytobiotic compounds can be absorbed in the small intestine [22,23]. Low absorption, biotransformation, and rapid excretion/clearance may lead to the low bioavailability of phytobiotics; this has been summarized in the review by Gessner et al [24]. First, most phytobiotics, especially polyphenols, form esters, glycosides, or polymers. For absorption in the small intestine, polyphenols have to be hydrolyzed to aglycones by either intestinal enzymes (lactase-phlorizin hydrolase and β-glucosidase) or microbial enzymes, as only the aglycone or glycoside forms of polyphenols can be absorbed in the small intestine [25, 26]. Second, phytobiotics have to be released from the plant matrix if they are administered as dry powder of plant parts, such as leaves, stems, and roots. However, this process may be limited by the lack of specific enzymes and a limited number of microbial enzymes in the intestine. Third, the phytobiotics and their metabolites are rapidly degraded within 2 to 12 h following absorption [22]. The absorbed phytobiotics are recognized as xenobiotics by the biotransformation system and undergo modifications, such as methylation, glucuronidation, and sulfation, in enterocytes and the liver [27,28]. These modifications render the phytobiotics water-soluble and allow their excretion in urine [29]. Fourth, the delivery of phytobiotics to the target tissues may be dependent on their binding affinity to albumin in the blood, which is based on the chemical structure of the phytobiotics [30,31]. This factor also influences the rate of clearance of phytobiotics from the blood. Moreover, it has been reported that long-term supplementation of polyphenols does not lead to their accumulation in plasma and tissues compared with single administration [32]. Several studies have investigated the bioavailability of phytobiotics in chickens. It has been reported that isoquinoline alkaloids, sanguinarine and dihydrosanguinarine, were found in the plasma at a concentration of 1 to 2 ng/mL between 5 min and 5 h post-treatment in broiler chickens orally administered Sangrovit (20 mg/kg) [33]. It has also been reported that quercetin and its metabolites were detected in plasma at a concentration of 0.04 to 0.14 ng/mL; however, the antioxidant capacity did not change in broiler chickens [34].

### INTESTINAL BARRIER FUNCTION AND GUT MICROBIOTA

These findings raise one important question, that is, how do phytobiotics lead to health benefits in animals when they have such a low bioavailability? Growing evidence suggests that phytobiotics may not have to be absorbed to exert their beneficial effects [35]. One study postulated that the non-absorbed fraction of phytobiotics may promote intestinal function or act as prebiotics [10]. The intestinal barrier system consists of several components, including, the mucus layer, immunoglobulin A, antibacterial peptides, and intercellular tight junctions (TJ) [36]. The mucosal layer plays an important role in the first line of defense against pathogen and toxin invasion in the gastrointestinal tract [37,38]. TJs are multiprotein junctional complexes of transmembrane proteins, such as claudin, occludin, and junctional adhesion molecule-A (JAM-A) and intracellular plaque proteins, such as zonula occludens (ZO). The gaps between the extracellular regions of the transmembrane proteins in the adjacent cells are involved in the paracellular passage of molecules in the intestinal lumen. Once the mucus layer and TJ barriers in the small intestine are destroyed, often by heat and overcrowding stress, glucocorticoid challenge, or high-fat diet [39–43], systemic and intestinal inflammation occurs, thereby triggering various chronic diseases through pathogen invasion [44].

Resveratrol, which is found in grapes, berries, peanuts, and red wine, is a well-known polyphenol, and extensive research has been conducted on this compound. Resveratrol supplementation has been reported to restore ZO-2, occludin, JAM-A, and claudin expression and to mitigate the increase in the level of plasma lipopolysaccharide (LPS)-binding protein, which is an indicator of intestinal barrier impairment [45]. One study using heat-stressed broiler chickens showed that resveratrol improved intestinal barrier function and mRNA expression of mucin-2, claudin-1, occludin, and E-cadherin [46]; attenuated nuclear factor-kappa B (NF-κB) protein expression; and induced the expression of epidermal growth factor mRNA in the intestine [47]. Another polyphenol, quercetin, which is found in onion, kale, and apples, also enhances intestinal barrier integrity by upregulating the level of claudin-4 [48], and by promoting the assembly of TJ proteins, ZO-2, occludin, and claudin-1 [49]. Naringenin is a flavanone that is rich in citrus fruits, and it increases the expression of occludin, JAM-A, and claudin-3 [50]. Moreover, it has been reported that a metabolite of phytobiotics produced by the gut microbiota is involved in intestinal barrier function. Urolithin A is a metabolite produced from ellagitannins and ellagic acid found in berries, grapes, and walnuts. It improves intestinal barrier integrity by inducing the expression of the TJ proteins, such as claudin-4, occludin, and ZO-1, via the activation of the aryl hydrocarbon receptor (AhR), and subsequently increasing binding to AhR nuclear factor erythroid 2–related factor 2 (Nrf2) in intestinal epithelial cells [51]. There is little information on the effects of polyphenols on the mucus layer, and one study has shown that grape pomace concentrate improves the villus height-to-crypt depth ratio, but does not affect the ileal mucin content [52]. In addition, the concentration of sialic acid, a constituent of mucin, decreased in broiler chickens fed grape extracts [53], which contain procyanidins as the main ingredient.

The effects of phytobiotics on the gut microbiota have also been extensively investigated, due to the importance of microbiota in the health and productivity of farm animals. The gut microbiota composition can affect growth parameters, such as growth rate, by influencing feed digestion and nutrient adsorption. The relationship between microbes, such as *Campylobacter*, *Escherichia coli*, *Lactobacilli*, and *Enterobacteria*, and the production performance has been extensively investigated (for more information, see the review by Iqbal et al [54]); however, this relationship has not yet been fully elucidated. It has been suggested that gut microbes metabolize phytobiotics into simpler metabolites to transform them into absorbable metabolites, whereas phytobiotics affect the population of gut microbes by interfering with their metabolic activities [54]. The transformation into simpler metabolites increases bioavailability and enhances the health-promoting effects in the intestine. Meanwhile, the prebiotic-like effects of phytobiotics suppress pathogenic bacteria, and this in turn improves the intestinal immune status and positively affects the population of beneficial bacteria. Several investigations on chickens have demonstrated the beneficial effects of plant constituents on the intestinal microbiota [46,52, 55,56].

## INTESTINAL AND SYSTEMIC INFLAMMATION

The intestinal epithelial barrier defends against the translocation of pathogenic bacteria and their harmful constituents into the circulation. The epithelial and immune cells present in the lamina propria recognize external substances, and they are activated to produce cytokines and other bioactive compounds to reinforce and restore the intestinal barrier. However, excessive protective responses may induce inflammation, resulting in barrier dysfunction. The NF-κB plays a key role in regulating inflammatory status. It is a transcriptional factor that is normally bound in an inactive state by inhibitory proteins in the cytosol. The release of NF-κB due to this inhibition is triggered by stimulation with cytokines, bacterial stimuli, and oxidants. This conformational change activates NF-κB for translocation into the nucleus, where it initiates the transcription of several genes involved in inflammation, including inflammatory cytokines, chemokines, inflammatory enzymes, adhesion molecules, and receptors (for more details see the review by Huang and Lee [57]). Mitogen-activated protein kinases (MAPKs) are a group of protein kinases that regulate cellular activities and activate another transcriptional factor, activator protein-1, which also induces the transcription of inflammatory genes [58]. Activated immune cells located near the epithelial cells secrete inflammatory cytokines, such as interleukin-6 (IL-6), interferon-γ, tumor necrosis factor-α (TNF-α), and inflammatory enzymes, such as inducible nitric oxide and cyclooxygenase. They cause the inflammation of intestinal epithelial cells and subsequently disrupt the intestinal barrier [59].

Phytobiotics may affect intestinal barrier functions, by not only upregulating the expression of the TJ proteins, but also influencing the intracellular signaling pathways inducing cytokine production [57,60]. Toll like receptors (TLRs) and nucleotide-binding oligomerization (NODs) are two primary targets of phytobiotics, and they can be activated by phytobiotics to inhibit the inflammation cascade. For example, it has been reported that curcumin inhibits TLR4 and NOD, whereas isothiocyanate inhibits TLR4 [61,62]. Resveratrol, epigallocatechin gallate, and quercetin do not inhibit TLR activation, but can suppress TLR4-mediated signal transduction by inhibiting TANK binding kinase 1 (TBK1), a kinase required for cytokine expression [63]. Moreover, Huang and Lee [57] proposed that carvacrol, curcumin, cinnamaldehyde, and thymol may inhibit or modulate the NF-κB and/or MAPK signaling pathways to mitigate inflammatory cascades, although their specific targets have not been identified. The detailed mechanisms governing the ameliorative effects of phytobiotics on inflammation have to be investigated in chickens.

LPS originating from the cell wall of gram-negative bacteria is an immune-stimulator that translocates into the circulation. It has negative effects on metabolism, physiology, and immunity. Abdominal or intravenous LPS injection has been extensively used as a non-microbial experimental model to investigate the effects of infection on metabolic dysfunction. LPS-stimulated inflammatory cytokine secretion induces oxidative stress, hepatic acute phase protein (APP) production, glucocorticoid secretion, muscle protein catabolism, and anorexia [64–68]. These symptoms alone or in combination induce growth retardation, anorexia, high mortality, and an increase in the feed conversion ratio (FCR). Chickens subjected to LPS challenge also showed an increase in the ratio of liver, spleen, and intestine weight to body weight [69]. Inflammation triggers alterations in metabolism that support the immune system, often involving the acceleration of skeletal muscle protein degradation. Cytokines such as IL-1, IL-6, TNF-α, and glucocorticoids participate in muscle proteolysis [70]. Gessner et al [24] proposed that amino acids donated from the degradation of muscle proteins and inhibition of muscle protein synthesis are used for APP synthesis and gluconeogenesis as energy fuel in the liver to counteract inflammation. The generation of these metabolites has been proposed as a metabolic cost [71,72]. If phytobiotics and their metabolites mitigate inflammatory status and subsequent protein degradation, amino acid utilization for such protein synthesis would no longer be necessary and normal muscle growth may progress. This could be one of the mechanisms that promote the growth performance of animals.

### Nrf2 PATHWAY AND DETOXIFYING SYSTEM

Phytobiotics have been traditionally viewed as antioxidants, and recently, it has been suggested that these compounds contribute to eliminating ROS, by not only direct antioxidant action, but also inducing the expression of antioxidant enzymes [73]. Nrf2 is a transcriptional factor that regulates the expression of antioxidant enzymes and proteins to protect cells against oxidative damage triggered by injury and inflammation [74]. Quercetin and resveratrol have been found to activate the Nrf2 pathway and induce the expression of ROS scavengers SOD and CAT [75]. In broiler chickens, LPS-induced intestinal oxidative stress was attenuated by quercetin via the activation of the Nrf2 pathway [76]. The activation of Nrf2 also inhibits NF-κB [75] and promotes the expression of peptide transporter 1 in intestinal cells [77]. It has also been shown that antagonistic crosstalk between sirtuin-1 (SIRT1) and NF-κB in the regulation of inflammation and metabolic disorders [78]. Several studies have proposed that polyphenols may protect against inflammation and metabolic diseases by enhancing the SIRT1 deacetylase activity [79]. Resveratrol was the first phenolic compound to activate SIRT1 [80]. However, this finding has been debated, and recent studies have shown that resveratrol is not a specific activator of SIRT1 [81]. These findings suggest that the Nrf2 pathway plays a role in the ameliorative effect of phytobiotics.

Phytobiotics are metabolized and excreted into the bile and urine in a similar fashion to xenobiotics in the intestine. Nuclear receptors are involved in the detoxification system, and the proteins in this system are regulated by transcription factors such as AhR and the pregnane X receptor (PXR). The binding of xenobiotic chemicals to these receptors induces the production of detoxifying enzymes, such as the cytochrome P450 family and glutathione-S-transferases in the liver and lungs, and this leads to the modification of phytobiotics to render them water-soluble to promote excretion in urine. It should be noted that the detoxification system interacts with the inflammatory network [82]. Xenoreceptors, PXR, and constitutive androstane receptors affect inflammation by interfering with NF-κB [83], and some phytobiotics have been shown to activate PXR and AhR [84]. Therefore, the involvement of the detoxification system in inflammatory signaling might be implicated in the ameliorative effects of phytobiotics.

### HOW DO PHYTOBIOTICS PROMOTE GROWTH?

Synthetic antibiotic/antimicrobial growth promoters (AGPs) have been used in meat production for several decades to increase productive parameters such as body weight gain and FCR [85]. However, the use of such growth promoters in animal production has been gradually restricted and prohibited in several countries. This has increased the interest in replacing these compounds with natural compounds that yield similar benefits. In this context, plant-derived compounds have emerged as alternatives to synthetic AGPs [21]. Numerous studies have reported the growth-promoting effects of phytobiotics, whereas the precise mechanisms underlying the role of phytobiotics as animal growth promoters have not yet been completely elucidated. Valenzuela-Grijalva et al [21] proposed four principal mechanisms by which phytobiotics may induce growth promotion: i) an improvement in feed status and feed consumption based on the flavor and palatability of the supplemented phytobiotics; ii) modulation of ruminal fermentation due to the antimicrobial effects; iii) an improvement in nutrient digestion and absorption with the augmentation of intestinal functions; and iv) direct and indirect anabolic activity on target tissues via the activation of endocrine and antioxidative defense systems. In the present review, I primarily focused on the immunomodulating effects of phytobiotics, which may be related to their health benefits and growth promotion effects.

**Figure 1:**
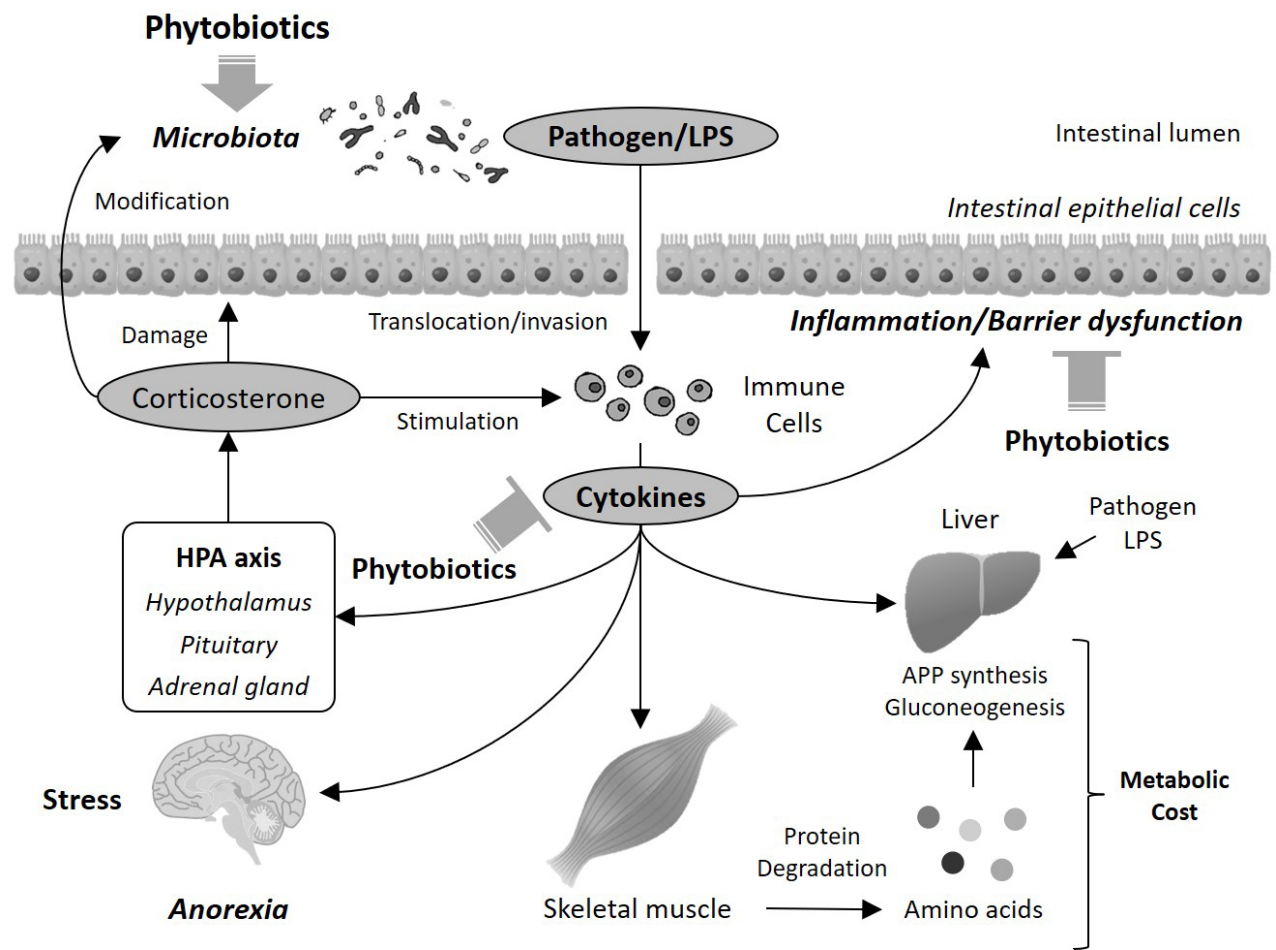
Possible mechanisms of mode of action in the beneficial effect of phytobiotics.

Considering these possible mechanisms, together with the findings regarding the improvement effects on intestinal condition and inflammatory status, it can be proposed that several beneficial effects of phytobiotics in animals may result from the improvement in intestinal function (Figure 1). Phytobiotics improve intestinal inflammatory status and barrier functions, possibly via the inhibition of TLRs and subsequent activation of NF-κB, the reduction in pathogenic bacteria, and the activation of the xenobiotics detoxifying system and Nrf2 pathway. This improvement in intestinal function subsequently prevents the translocation of pathogens and harmful constituents such as LPS into the circulatory system, and induction of systemic inflammation via excess secretion of cytokines and glucocorticoids. In this way, metabolism is normalized to reduce metabolic expenditure. These changes may be involved in growth promotion in animals. It is also possible that the central nervous system and hypothalamic-pituitary-adrenal (HPA) axis could participate in this scenario. The HPA axis controls glucocorticoid secretion, and excess and long-term secretion of glucocorticoids disrupts the intestinal barrier function and the intestinal microbiota [86]. These disruptions accelerate cytokine production, which in turn stimulates the HPA axis to secrete glucocorticoids. In this way, a vicious cycle is initiated, which exacerbates inflammatory and metabolic dysregulation. Additionally, LPS in the circulation induces not only inflammation but also anorexia in chickens [66]. These findings suggest that the improvement in intestinal function may play an important role in the growth-promoting effect of phytobiotics.

### SUMMARY AND PERSPECTIVE

There is an increasing desire to replace synthetic AGPs in animal production with safer natural compounds to avoid the increasing risk of antimicrobial resistance. The health benefits and growth-promoting effects of phytobiotics may be dependent on several mechanisms based on their various biological activities. However, the mode of action is likely to be consistent despite the supplemental concentration and form (powder/oil), the age at and duration of administration, strain, and sex. Moreover, it is difficult to determine the precise mechanism of action of each phytobiotic, as they exist in plants as different mixtures. This may also be a reason for the differences in effects among various investigations. Advances in the knowledge of the effects of phytobiotics on intestinal function and subsequent metabolic changes and inflammation could contribute to a further understanding of the use of phytobiotics in animal production.

## References

[1] Liu RH (2004). Potential synergy of phytochemicals in cancer prevention: mechanism of action. J Nutr.

[2] Shimao R, Muroi H, Furukawa K, Toyomizu M, Kikusato M (2019). Effects of low-dose oleuropein diet supplementation on the oxidative status of skeletal muscles and plasma hormonal concentration of growing broiler chickens. Br Poult Sci.

[3] Kikusato M, Xue G, Pastor A, Niewold TA, Toyomizu M (2021). Effects of plant-derived isoquinoline alkaloids on growth performance and intestinal function of broiler chickens under heat stress. Poult Sci.

[4] Kim DK, Lillehoj HS, Lee SH, Jang SI, Lillehoj EP, Bravo D (2013). Dietary *Curcuma longa* enhances resistance against *Eimeria maxima* and *Eimeria tenella* infections in chickens. Poult Sci.

[5] Olson JB, Ward NE, Koutsos EA (2008). Lycopene incorporation into egg yolk and effects on laying hen immune function. Poult Sci.

[6] Tsao R, Deng Z (2004). Separation procedures for naturally occurring antioxidant phytochemicals. J Chromatogr B Analyt Technol Biomed Life Sci.

[7] Lee MT, Lin WC, Yu B, Lee TT (2017). Antioxidant capacity of phytochemicals and their potential effects on oxidative status in animals - a review. Asian-Australas J Anim Sci.

[8] Perron NR, Brumaghim JL (2009). A review of the antioxidant mechanisms of polyphenol compounds related to iron binding. Cell Biochem Biophys.

[9] Halliwell B, Rafter J, Jenner A (2005). Health promotion by flavonoids, tocopherols, tocotrienols, and other phenols: direct or indirect effects? Antioxidant or not?. Am J Clin Nutr.

[10] Martel J, Ojcius DM, Ko YF, Young JD (2020). Phytochemicals as prebiotics and biological stress inducers. Trends Biochem Sci.

[11] Williamson G, Kay CD, Crozier A (2018). The bioavailability, transport, and bioactivity of dietary flavonoids: a review from a historical perspective. Compr Rev Food Sci Food Saf.

[12] Pandey AK, Kumar P, Saxena MJ, Gupta RC, Srivastava A, Lall R (2019). Feed additives in animal health. Nutraceuticals in veterinary medicine.

[13] Qin S, Hou DX (2017). The biofunctions of phytochemicals and their applications in farm animals: the Nrf2/Keap1 system as a target. Engineering.

[14] AL-Sagan AA, Khalil S, Hussein EOS, Attia YA (2020). Effects of fennel seed powder supplementation on growth performance, carcass characteristics, meat quality, and economic efficiency of broilers under thermoneutral and chronic heat stress conditions. Animals.

[15] Starčević K, Krstulović L, Brozić D (2015). Production performance, meat composition and oxidative susceptibility in broiler chicken fed with different phenolic compounds. J Sci Food Agric.

[16] Cayan H, Erener G (2015). Effect of olive leaf (*Olea europaea*) powder on laying hens performance, egg quality and egg yolk cholesterol levels. Asian-Australas J Anim Sci.

[17] Attia YA, Bakhashwain AA, Bertu NK (2017). Thyme oil (*Thyme vulgaris L*.) as a natural growth promoter for broiler chickens reared under hot climate. Ital J Anim Sci.

[18] Liu ZY, Wang XL, Ou SQ, Hou DX, He JH (2020). Sanguinarine modulate gut microbiome and intestinal morphology to enhance growth performance in broilers. PLoS One.

[19] Aljumaah MR, Suliman GM, Abdullatif AA, Abudabos AM (2020). Effects of phytobiotic feed additives on growth traits, blood biochemistry, and meat characteristics of broiler chickens exposed to *Salmonella typhimurium*. Poult Sci.

[20] Lillehoj H, Liu Y, Calsamiglia S (2018). Phytochemicals as antibiotic alternatives to promote growth and enhance host health. Vet Res.

[21] Valenzuela-Grijalva NV, Pinelli-Saavedra A, Muhlia-Almazan A, Domínguez-Díaz D, González-Ríos H (2017). Dietary inclusion effects of phytochemicals as growth promoters in animal production. J Anim Sci Technol.

[22] Manach C, Williamson G, Morand C, Scalbert A, Rémésy C (2005). Bioavailability and bioefficacy of polyphenols in humans. I. Review of 97 bioavailability studies. Am J Clin Nutr.

[23] Teng Z, Yuan C, Zhang F (2012). Intestinal absorption and first-pass metabolism of polyphenol compounds in rat and their transport dynamics in Caco-2 cells. PLoS One.

[24] Gessner DK, Ringseis R, Eder K (2017). Potential of plant polyphenols to combat oxidative stress and inflammatory processes in farm animals. J Anim Physiol Anim Nutr.

[25] Murota K, Shimizu S, Miyamoto S (2002). Unique uptake and transport of isoflavone aglycones by human intestinal caco-2 cells: comparison of isoflavonoids and flavonoids. J Nutr.

[26] Manach C, Scalbert A, Morand C, Rémésy C, Jiménez L (2004). Polyphenols: food sources and bioavailability. Am J Clin Nutr.

[27] Spencer JPE (2003). Metabolism of tea flavonoids in the gastrointestinal tract. J Nutr.

[28] Scalbert A, Morand C, Manach C, Rémésy C (2002). Absorption and metabolism of polyphenols in the gut and impact on health. Biomed Pharmacother.

[29] Holst B, Williamson G (2008). Nutrients and phytochemicals: from bioavailability to bioefficacy beyond antioxidants. Curr Opin Biotechnol.

[30] Liu EH, Qi LW, Li P (2010). Structural relationship and binding mechanisms of five flavonoids with bovine serum albumin. Molecules.

[31] Dangles O, Dufour C, Manach C, Morand C, Remesy C (2001). Binding of flavonoids to plasma proteins. Methods Enzymol.

[32] Bieger J, Cermak R, Blank R (2008). Tissue distribution of quercetin in pigs after long-term dietary supplementation. J Nutr.

[33] Hu NX, Chen M, Liu YS (2019). Pharmacokinetics of sanguinarine, chelerythrine, and their metabolites in broiler chickens following oral and intravenous administration. J Vet Pharmacol Ther.

[34] Rupasinghe HV, Ronalds CM, Rathgeber B, Robinson RA (2010). Absorption and tissue distribution of dietary quercetin and quercetin glycosides of apple skin in broiler chickens. J Sci Food Agric.

[35] Wu XM, Tan RX (2019). Interaction between gut microbiota and ethnomedicine constituents. Nat Prod Rep.

[36] Suzuki T (2020). Regulation of the intestinal barrier by nutrients: the role of tight junctions. Anim Sci J.

[37] Quintana-Hayashi MP, Padra M, Padra JT, Benktander J, Lindén SK (2018). Mucus-pathogen interactions in the gastrointestinal tract of farmed animals. Microorganisms.

[38] Capaldo CT, Powell DN, Kalman D (2017). Layered defense: how mucus and tight junctions seal the intestinal barrier. J Mol Med.

[39] Vicuña EA, Kuttappan VA, Galarza-Seeber R (2015). Effect of dexamethasone in feed on intestinal permeability, differential white blood cell counts, and immune organs in broiler chicks. Poult Sci.

[40] Nanto-Hara F, Kikusato M, Ohwada S, Toyomizu M (2020). Heat stress directly affects intestinal integrity in broiler chickens. J Poult Sci.

[41] Alhenaky A, Abdelqader A, Abuajamieh M, Al-Fataftah AR (2017). The effect of heat stress on intestinal integrity and *Salmonella* invasion in broiler birds. J Therm Biol.

[42] Kridtayopas C, Rakangtong C, Bunchasak C, Loongyai W (2019). Effect of prebiotic and synbiotic supplementation in diet on growth performance, small intestinal morphology, stress, and bacterial population under high stocking density condition of broiler chickens. Poult Sci.

[43] Murakami Y, Tanabe S, Suzuki T (2016). High-fat diet-induced intestinal hyperpermeability is associated with increased bile acids in the large intestine of mice. J Food Sci.

[44] Manco M, Putignani L, Bottazzo GF (2010). Gut microbiota, lipopolysaccharides, and innate immunity in the pathogenesis of obesity and cardiovascular risk. Endocr Rev.

[45] Mayangsari Y, Suzuki T (2018). Resveratrol ameliorates intestinal barrier defects and inflammation in colitic mice and intestinal cells. J Agric Food Chem.

[46] Zhang C, Zhao XH, Yang L (2017). Resveratrol alleviates heat stress-induced impairment of intestinal morphology, microflora, and barrier integrity in broilers. Poult Sci.

[47] Liu L, Fu C, Yan M (2016). Resveratrol modulates intestinal morphology and HSP70/90, NF-κB and EGF expression in the jejunal mucosa of black-boned chickens on exposure to circular heat stress. Food Funct.

[48] Amasheh M, Schlichter S, Amasheh S (2008). Quercetin enhances epithelial barrier function and increases claudin-4 expression in Caco-2 cells. J Nutr.

[49] Suzuki T, Hara H (2009). Quercetin enhances intestinal barrier function through the assembly of zonnula occludens-2, occludin, and claudin-1 and the expression of claudin-4 in Caco-2 cells. J Nutr.

[50] Azuma T, Shigeshiro M, Kodama M, Tanabe S, Suzuki T (2013). Supplemental naringenin prevents intestinal barrier defects and inflammation in colitic mice. J Nutr.

[51] Singh R, Chandrashekharappa S, Bodduluri SR (2019). Enhancement of the gut barrier integrity by a microbial metabolite through the Nrf2 pathway. Nat Commun.

[52] Viveros A, Chamorro S, Pizarro M, Arija I, Centeno C, Brenes A (2011). Effects of dietary polyphenol-rich grape products on intestinal microflora and gut morphology in broiler chicks. Poult Sci.

[53] Chamorro S, Romero C, Brenes A (2019). Impact of a sustained consumption of grape extract on digestion, gut microbial metabolism and intestinal barrier in broiler chickens. Food Funct.

[54] Iqbal Y, Cottrell JJ, Suleria HAR, Dunshea FR (2020). Gut microbiota-polyphenol interactions in chicken: a review. Animals.

[55] Lee KW, Kim JS, Oh ST, Kang CW, An BK (2015). Effects of dietary sanguinarine on growth performance, relative organ weight, cecal microflora, serum cholesterol level and meat quality in broiler chickens. J Poult Sci.

[56] Abu Hafsa SH, Ibrahim SA (2018). Effect of dietary polyphenol-rich grape seed on growth performance, antioxidant capacity and ileal microflora in broiler chicks. J Anim Physiol Anim Nutr.

[57] Huang CM, Lee TT (2018). Immunomodulatory effects of phytogenics in chickens and pigs - a review. Asian-Australas J Anim Sci.

[58] Wang A, Al-Kuhlani M, Johnston SC, Ojcius DM, Chou J, Dean D (2013). Transcription factor complex AP-1 mediates inflammation initiated by *Chlamydia pneumoniae* infection. Cell Microbiol.

[59] Awad WA, Hess C, Hess M (2017). Enteric pathogens and their toxin-induced disruption of the intestinal barrier through alteration of tight junctions in chickens. Toxins.

[60] Shimizu M (2017). Multifunctions of dietary polyphenols in the regulation of intestinal inflammation. J Food Drug Anal.

[61] Huang S, Zhao L, Kim K, Lee DS, Hwang DH (2008). Inhibition of Nod2 signaling and target gene expression by curcumin. Mol Pharmacol.

[62] Shibata T, Nakashima F, Honda K (2014). Toll-like receptors as a target of food-derived anti-inflammatory compounds. J Biol Chem.

[63] Youn HS, Lee JY, Fitzgerald KA, Young HA, Akira S, Hwang DH (2005). Specific inhibition of MyD88-independent signaling pathways of TLR3 and TLR4 by resveratrol: molecular targets are TBK1 and RIP1 in TRIF complex. J Immunol.

[64] Frost RA, Lang CH (2008). Regulation of muscle growth by pathogen-associated molecules. J Anim Sci.

[65] Zheng YW, Zhang JY, Zhou HB (2020). Effects of dietary pyrroloquinoline quinone disodium supplementation on inflammatory responses, oxidative stress, and intestinal morphology in broiler chickens challenged with lipopolysaccharide. Poult Sci.

[66] Tachibana T, Kodama T, Yamane S, Makino R, Khan SI, Cline MA (2017). Possible role of central interleukins on the anorexigenic effect of lipopolysaccharide in chicks. Br Poult Sci.

[67] Han H, Zhang J, Chen Y (2020). Dietary taurine supplementation attenuates lipopolysaccharide-induced inflammatory responses and oxidative stress of broiler chickens at an early age. J Anim Sci.

[68] Horvatić A, Guillemin N, Kaab H (2018). Integrated dataset on acute phase protein response in chicken challenged with *Escherichia coli* lipopolysaccharide endotoxin. Data Brief.

[69] Roura E, Homedes J, Klasing KC (1992). Prevention of immunologic stress contributes to the growth-permitting ability of dietary antibiotics in chicks. J Nutr.

[70] Zhou J, Liu B, Liang C, Li Y, Song YH (2016). Cytokine signaling in skeletal muscle wasting. Trends Endocrinol Metab.

[71] Niewold TA (2007). The nonantibiotic anti-inflammatory effect of antimicrobial growth promoters, the real mode of action? A hypothesis. Poult Sci.

[72] Broom LJ, Kogut MH (2018). Inflammation: friend or foe for animal production?. Poult Sci.

[73] Christensen LP, Christensen KB, Watson RR, Preedy VR, Zibadi S (2014). The role of direct and indirect polyphenolic antioxidants in protection against oxidative stress. Polyphenols in human health and disease.

[74] Kim J, Cha YN, Surh YJ (2010). A protective role of nuclear factor-erythroid 2-related factor-2 (Nrf2) in inflammatory disorders. Mutat Res.

[75] Qin S, Hou DX (2016). Multiple regulations of Keap1/Nrf2 system by dietary phytochemicals. Mol Nutr Food Res.

[76] Sun L, Xu G, Dong Y, Li M, Yang L, Lu W (2020). Quercetin protects against lipopolysaccharide-induced intestinal oxidative stress in broiler chickens through activation of Nrf2 pathway. Molecules.

[77] Geillinger KE, Kipp AP, Schink K, Roder PV, Spanier B, Daniel H (2014). Nrf2 regulates the expression of the peptide transporter PEPT1 in the human colon carcinoma cell line Caco-2. Biochim Biophys Acta.

[78] Kauppinen A, Suuronen T, Ojala J, Kaarniranta K, Salminen A (2013). Antagonistic crosstalk between NF-κB and SIRT1 in the regulation of inflammation and metabolic disorders. Cell Signal.

[79] Chung S, Yao H, Caito S, Hwang J, Arunachalam G, Rahman I (2010). Regulation of SIRT1 in cellular functions: role of polyphenols. Arch Biochem Biophys.

[80] Baur JA, Pearson KJ, Price NL (2006). Resveratrol improves health and survival of mice on a high-calorie diet. Nature.

[81] Beher D, Wu J, Cumine S (2009). Resveratrol is not a direct activator of SIRT1 enzyme activity. Chem Biol Drug Des.

[82] Xie W, Tian Y (2006). Xenobiotic receptor meets NF-κB, a collision in the small bowel. Cell Metab.

[83] Moreau A, Vilarem MJ, Maurel P, Pascussi JM (2008). Xenoreceptors CAR and PXR activation and consequences on lipid metabolism, glucose homeostasis, and inflammatory response. Mol Pharm.

[84] Satsu H, Hiura Y, Mochizuki K, Hamada M, Shimizu M (2008). Activation of pregnane X receptor and induction of MDR1 by dietary phytochemicals. J Agric Food Chem.

[85] Hao H, Cheng G, Iqbal Z (2014). Benefits and risks of antimicrobial use in food-producing animals. Front Microbiol.

[86] Misiak B, Łoniewski I, Marlicz W (2020). The HPA axis dysregulation in severe mental illness: can we shift the blame to gut microbiota?. Prog Neuropsychopharmacol Biol Psychiatry.

